# Dynamic copay health plans, AI-enabled price transparency, and member experience

**DOI:** 10.1093/haschl/qxag139

**Published:** 2026-06-16

**Authors:** Arindam Debbarma, Bryan E Dowd

**Affiliations:** Walmart Inc, Bentonville, AR 72712, United States; Division of Health Policy and Management, School of Public Health, University of Minnesota, Minneapolis, MN 55455, United States

**Keywords:** health insurance benefit design, dynamic copay, variable copay, out-of-pocket expenditure, tiered plan, reference pricing, care coordination, value-based care, Surest, AI

## Abstract

Employers and enrollees face premium growth and rising cost sharing, yet patients often don’t know what they will owe when they seek care. Traditional “consumerism” has struggled because quality is hard to observe, prices are difficult to estimate in real time, and many services are urgent. We review the evolution of employer plan design, from broad network Preferred Provider Organizations (PPOs) to narrow and tiered networks, and reference pricing to the new emerging “dynamic copay” plans. These plans translate negotiated price variation into provider- and service-specific dollar copays displayed pre-service, often through app-based tools enabled by Centers for Medicare & Medicaid Services regulations, Transparency in Coverage data and AI-enabled analytics. Using UnitedHealthcare's Surest plan as an illustrative case, we examine the operational pre-requisites for “copay integrity,” likely effects on out-of-pocket (OOP) predictability and spending in light of peer-reviewed evidence on tiered designs, and the constraints imposed by health insurance literacy, the limited share of spending that is meaningfully shoppable below the OOP maximum, and the long-run attenuation of steering effects. We conclude by proposing that pairing dynamic copays with reference pricing, layered onto a tiered network, may better address these limitations by strengthening steerage, improving OOP predictability and aligning member shopping incentives with higher-value care.

## Introduction

According to the Centers for Medicare & Medicaid Services (CMS), national healthcare spending grew 7.2% from 2023 to 2024, reaching $15 474 per person.^1^ Health insurance covers most of that spending, and rising costs translate into higher premiums: the 2025 Kaiser Family Foundation (KFF) employer survey reports an average annual family premium above $25 500.^2^ Although employers nominally pay most of that amount, economic theory and empirical work consistently find that workers ultimately bear the cost through lower wages or slower wage growth.^3-5^

Out-of-pocket (OOP) exposure has risen alongside premiums. The average single-coverage deductible reached $1886 in 2025, up 42% since 2015, and 21% of covered workers face OOP maximums above $6000.^2^ Two-thirds of adults in a recent KFF poll reported anxiety about affording care.^6^ Beyond affordability, many households face “unpredictable exposure,” the inability to know, at the point of decision, what they will ultimately owe—because liability depends on benefit design, provider choice, and negotiated prices that remain opaque until claims are adjudicated.

Employers and insurers have responded with successive generations of plan design intended to steer members toward lower-priced or higher-value providers: narrow networks, tiered networks, reference pricing, high-deductible health plans (HDHPs), and value-based insurance design (VBID).^7-9^ In 2025, 24% of firms with 5000 or more workers offered a tiered or high-performance network in their largest plan.^2^ Each of these designs has accumulated a peer-reviewed evidence base on its effects, and a parallel literature has documented the behavioral limits of consumer-directed approaches, poor shopping behavior even among well-compensated enrollees, low use of price transparency tools, and persistent ambiguity about what services actually cost.^10-12^

Against this backdrop, “dynamic copay” plans—also marketed as “variable copay” or “copay-only” designs—have begun to attract attention. These products replace deductibles and coinsurance with fixed dollar copays that vary across providers, locations, and services and are surfaced to members before care is scheduled, typically through a mobile or web application. The label “dynamic” is misleading; copays are not estimated in real time or personalized at the member level but rather set at the practitioner-location-service level and refreshed when contracts or networks change.^13^

In this paper, we argue that though the new “dynamic copay” plans attempt to address a failure of consumer driven designs (unpredictable out-of-pocket exposure), they might not be able to address issues related to poor care coordination, prescription of low-value care, or align incentives around total cost of care, and they may shift the distribution of financial risk between premiums and point-of-care payments that warrant careful policy analysis.

## Steering in plan design: tiered networks, reference pricing, and VBID

The mechanism of varying fixed-dollar member cost-sharing across in-network providers as a steering tool is not new. Tiered networks were introduced in the early 2000s as a less restrictive alternative to narrow networks: instead of excluding providers, plans stratified in-network providers by price, total cost of care, and quality, and assigned differential copays.^14^ Long-running examples include the Massachusetts Group Insurance Commission, BlueCross BlueShield of Massachusetts plans, and the Minnesota State Employee Group Insurance Program (SEGIP), the last of which has used clinic-level total-cost-of-care tiers for primary care for over a decade and has reported sustained migration of members toward lower-tier clinics.^15,16^ Empirical evidence on tiered designs is broadly consistent. Prager found that tiered hospital networks reduced expected hospital spending by 8%-17%.^17^ Ackley reported per-episode spending 4.5%-6.3% lower under tiered designs than HDHPs with coinsurance.^18^ Frank et al. observed a 7.6% point shift in hospital admissions toward preferred-tier hospitals.^19^ Sinaiko and Rosenthal estimated that bottom-tier specialists lost 11%-12% of their new-patient share.^20^ Sinaiko, Landrum, and Chernew found that enrollment in a tiered-physician plan reduced overall medical spending by approximately 5%.^21^ A systematic review concluded that tiered networks produce modest but real steerage, with mixed effects on access and equity.^22^

Reference pricing extends the same logic by setting a maximum plan contribution for a defined service, with members liable for any amount above the reference price. CalPERS' implementation for orthopedic procedures redirected volume toward designated facilities and reduced negotiated prices.^23^ Whaley and Brown found that reference pricing for surgical services prompted firm-side responses—price reductions among non-designated providers—and altered the choice architecture facing consumers.^24^ VBID, by contrast, varies cost-sharing on clinical rather than price grounds, lowering copays for high-value services and medications independently of provider efficiency.^25^ Dynamic copays can be VBID-consistent if the copay schedule incorporates quality and value alongside price, but the underlying mechanism is salience and granularity rather than clinical nuance.

From the literature discussed above, two qualifications shape any reasonable expectation for dynamic copays. First, steering effects can attenuate over time, Prager et al. found no measurable steering effect for a tiered specialist-physician plan after a decade.^26^ Second, copay differentials must be large to overcome trust in clinician recommendations; Sinaiko documented that recommendations from trusted clinicians dominated copay differentials below approximately $300.^27^ Literature in similar settings has reached compatible conclusions, including that tiered cost-sharing for primary care gatekeeper clinics produces clinic-level price reductions but that primary care clinic leaders themselves report meaningful operational barriers to delivering on plan-design value goals^28-30^(see Table 1).

**Table 1.** Traditional employer plan design strategies and member-facing price signals.

Plan design	Primary mechanism	What the member “sees” at the point of care	Member-experience implications (price transparency & usability)
Broad-network PPO	Negotiated rates with deductibles/coinsurance; broad provider choice	Deductible in addition to copays/coinsurance; limited upfront “dollar price”	Low pre-service predictability for many services; cost is often a function of deductible status + billed components rather than an easily comparable price
Narrow networks	Selective contracting to lower unit prices	In network and out of network status; specialists without referrals	Can reduce premiums but may constrain access and does not necessarily create actionable pre-service OOP prices within the network
Tiered/high-performance networks	Differential incentives and cost of care across tiers of in-network providers	Lower copays/coinsurance for lower tiers	More choice than narrow networks; however, tiers can be restrictive and lack clear, pre-service OOP comparability
Reference pricing	Employer sets a maximum plan contribution for a service	“Pay the difference” above the reference price	Strong incentive signal but can generate billing complexity and member confusion about what counts in the service
Dynamic copay	Translate negotiated price variation into provider- and service-specific copays shown pre-service	Upfront dollar copays that vary by provider/service	Can improve predictability and comparability at the decision point, but depends on accurate provider/service matching and adjudication alignment (“copay integrity”)

## What is new about “dynamic copay” models?

Against this background, the incremental contribution of dynamic copay designs can be stated precisely. *Copay Granularity:* instead of having 2 or 3 tier categories, copays vary at the provider-location-service level and can reflect continuous variation in negotiated rates and value scores.^13^  *Point-of-service salience:* members see a fixed dollar amount before the encounter rather than computing percentage coinsurance against a future allowed amount, reducing cognitive load at the decision point. *Transparency-data plumbing:* the federal Hospital Price Transparency rule (effective January 2021) and the Transparency in Coverage (TiC) rule (effective July 2022) require machine-readable disclosure of negotiated rates and member-facing self-service estimators, making it operationally feasible to assemble and refresh the underlying price data at scale.^31-33^

In UnitedHealthcare's Surest plan, deductibles and coinsurance are eliminated; members compare providers by copay through an application before scheduling; provider-facing materials describe enhanced electronic data interchange transactions through which contracted “variable copay” amounts are returned at the practitioner-location level.^34,35^ Surest is, in this sense, an evolution of long-studied steering designs–relying on price salience and granularity rather than network restriction or post-adjudication assessment.

This framing matters because it bounds expectations. Dynamic copays do not constitute a new theory of consumer behavior. They inherit the behavioral and structural limits documented in the tiered-network and reference-pricing literatures and add operational complexity in exchange for finer resolution and earlier salience. Whether that trade improves member experience, spending, or equity is an empirical question that the existing evidence base is not yet positioned to answer (Table 2 compares the two designs across these dimensions).

**Table 2.** Tiered networks vs dynamic copay designs: implications for granularity, predictability, and the “shopping” experience.

Design dimension	Tiered networks	Dynamic copay (“variable copay”) plans	Why it matters for member experience
Provider inclusion	Most providers remain in-network; differentiated by tier	Providers remain in-network; differentiated by copay level (often more continuous)	Dynamic copays can create steerage without excluding providers, relying on price salience rather than network restriction but might not reduce total cost of care
Incentive granularity	Coarse categories (Tier 1/Tier 2…)	Provider- and service-specific dollar copays	Finer granularity can make differences more actionable, especially where prices vary widely within a tier
Explanation of cost sharing	Copays/coinsurance by tier; deductible/coinsurance often still applies	Often copay-only	Dollar copays reduce math and cognitive load vs percent coinsurance
Pre-service price visibility	Member may know tier but not liability if coinsurance applies	Member sees a dollar amount pre-service (when matching is accurate)	Predictability improves only when the amount displayed is decision-ready and clearly defined
Operational requirement	Accurate tier attribution with directory	High-fidelity provider/location matching, service translation, adjudication alignment	Dynamic copays shift complexity to the plan/vendor; success depends on “last mile” execution
Compatibility with other steering tools	Can pair with reference pricing	Can be layered on tiering and/or paired with reference pricing	Combining tools may strengthen steerage, but complexity increases if member-facing rules are unclear

## AI, price transparency data, and the “last mile” problem

To meet federal transparency requirements, hospitals must post a comprehensive machine-readable file of “standard charges” and a consumer-friendly set of shoppable services.^31^ Under the TiC regulations, most group health plans and issuers must provide real-time, personalized cost-sharing estimates through an internet-based self-service tool.^33^ CMS itself has acknowledged that disclosure is not the same as usable pricing.^32^ The files are expressed in billing primitives—codes, modifiers, place of service and separate facility/professional components—while members search for everyday descriptions. Translating raw files into accurate member-facing estimates requires reconciling code clusters, ancillary components, and facility fees that often differ from the headline service. The plausible roles of AI in this last-mile problem are bounded but real. Natural-language models can map consumer queries to billing codes or service bundles and flag ambiguities. Analytic tools can predict common ancillary components and flag services with historically high variance. Recommender systems can incorporate quality and access constraints. Conversational interfaces can explain why prices differ across sites-of-care. Equally critical is a consideration of the inherent limitations of AI. Service-definition fuzziness is a structural feature of medical billing, not an artifact of presentation. For a given clinician, the correlation of cost-sharing across a cluster of related Current Procedural Terminology (CPT) codes can be empirically variable, so even members shopping in good faith may face hidden incidental variance when actual coding diverges from the headline service. AI cannot resolve uncertainty that is irreducible at the time of decision; it can at best surface ranges and flag risk. Trust and clinician relationships dominate price signals at clinically meaningful magnitudes, as Sinaiko's work indicates.^27^

Because AI-enabled tools steer utilization and shape OOP estimates, they function as decision-support systems and require corresponding governance—bias and disparate-impact testing, explainability and version control, and protections aligned with HIPAA's minimum-necessary principle and the Security Rule.^36^ The NIST AI Risk Management Framework and its generative AI profile provide a useful structure for documenting intended use, measuring reliability, and managing drift as contracts and networks change.^37^ Without these guardrails, the risk is that AI obscures rather than clarifies the underlying uncertainty.

## Behavioral foundations, the shoppable share, and cost-sharing sensitivity

The case for dynamic copays rests on assumptions that behavioral economics and consumer-directed-plan literatures challenge. Brot-Goldberg et al., 2017 found that highly compensated employees at a large, self-insured firm responded to a switch into a high-deductible plan primarily by reducing utilization across the board rather than by shopping on price; the entire 11.8%-13.8% spending reduction reflected quantity reductions rather than substitution toward lower-priced providers.^10^ Even consumers with substantial financial exposure and access to price information did not shop. Anderson et al. in 2024 documented a complementary pattern in spending around the arrival of medical bills; households increase spending by approximately 22% after a scheduled service and then reduce spending by roughly 11% once the bill arrives, with bill effects strongest where pricing information is most salient—evidence that consumers face significant ex ante price uncertainty and update their behavior only when prices are revealed ex post.^12^

Dynamic copays attempt to move that information forward in time, which is a real improvement. But the underlying behavioral economics remains—members must allocate cognitive effort to shopping, weigh trade-offs across uncertain dimensions (price, quality, access, continuity, network), and act against the gravitational pull of trusted clinician recommendations. Survey work suggests that few enrollees use price transparency tools even when available, and that the threshold for action is high. Sinaiko's earlier survey work in a tiered-network setting found that recommendations from trusted clinicians dominated copay differentials below approximately $300, even among members who valued price information.^27^ More broadly, health insurance literacy is a documented barrier to effective consumer-directed care; members struggle to translate benefit rules into expected costs, and the fluency required to operate a dynamic-copay app at the point of decision is unlikely to be uniformly distributed across the population. Designs that depend on member sophistication risk widening rather than narrowing equity gaps.

The shoppable-share question further bounds the design. Estimates of the share of healthcare spending that is “shoppable” (ie, schedulable in advance) commonly fall around 40%.^38^ However, the relevant share for dynamic copay design is narrower—care that is both shoppable and incurred by members still below their out-of-pocket maximum (MOOP). Members who have hit MOOP face zero marginal cost for in-network services, while a small share of high-cost members account for a disproportionate share of total spending.^7^ Because dynamic copay plans typically eliminate the deductible and coinsurance, the member's marginal price is the displayed copay across the entire range from first dollar through MOOP—a flatter cost-sharing schedule than the deductible-plus-coinsurance structure of a traditional PPO, in which sensitivity changes as a member accumulates spending toward the deductible and the MOOP. By construction, dynamic copays exert their leverage primarily on the moderate-utilization middle: members with enough plannable care to face copay differences but not so much that they have already exhausted cost-sharing exposure. This bounds the design's plausible spending impact.

A related question is whether the individual is the right unit of steering at all. Members in this middle of the distribution have limited experience with any single service, may lack the basis to differentiate value, and do not drive the bulk of total spending. Aligning incentives at the provider or clinical-group level—through gain-sharing, value-based contracts, capitation, or shared-savings arrangements—may achieve more durable spending reductions than relying on individual point-of-decision shopping. The architecture of three-way gain-sharing, in which realized savings from value-based contracting are split among patients, providers, and payers, aligns incentives at the level where decisions about treatment intensity and site of care are actually made. Member-facing copays are most useful at the margin, helping patients distinguish among comparable in-network options for plannable services; they are not a substitute for the supply-side contracting that determines the bulk of per-episode cost. We view dynamic copays as complementary to such supply-side levers rather than as substitutes.

## Expected impacts on premiums, out-of-pocket costs, and member experience

Dynamic copay designs address how members experience cost sharing. Instead of asking members to translate negotiated prices and benefit math into a personal cost estimate, these products aim to present a clear, fixed dollar amount for a given provider and service before care is scheduled. In theory, that last-mile translation can change both where and when members seek care and how they experience cost sharing.

### Health premiums and total spending: competing pathways

Potential downward pressure on premiums or employer costs comes primarily from steerage. If variable copays are calibrated to make high-priced or low-quality sites of care more expensive for members, some share of “shoppable” services should migrate to lower-priced care sites. The mechanism shares the underlying logic of tiered networks and reference pricing but reduces friction by presenting a simple dollar comparison at the point of decision. The magnitude of potential savings is bounded by how much is shoppable—approximately 40% of total healthcare spending—and narrower once one conditions on members who are still cost-sharing sensitive below their OOP maximum.^39^

The most useful empirical anchor for expected magnitudes comes from the peer-reviewed tiered-network literature reviewed above, which documents 5%-17% spending reductions or volume shifts under designs of similar mechanism.^17-22^ Plan-commissioned analysis of Surest by Aon is directionally consistent, reporting $365 lower total allowed spend per member per year in 2021 and $412 in 2022 relative to a matched control group, with total cost of care approximately 7.5%-7.7% lower than controls.^40^ Aon's estimates should be interpreted with caveats—the analysis is plan-commissioned, focuses on allowed claims rather than premiums or net plan cost, and applies to a specific product implementation. The tiered-network literature further cautions that steering effects can attenuate over time and that copay differentials below approximately $300 often fail to overcome trust in clinician recommendations.^26,27^

At minimum, dynamic copay designs require (1) a constantly updated provider directory with accurate network status and location data, (2) negotiated rate files that can be linked to providers and settings, and (3) an engine that can map a member's need into services. While this technology enablement adds administrative costs, a second-order pathway operates through utilization and mix. Copay-only structures may reduce financial barriers for primary and preventive care, potentially improving downstream outcomes, but they can also increase use of discretionary care if copays are set too low relative to the marginal cost of additional utilization. The policy question is not whether utilization rises or falls, but whether utilization shifts toward higher-value settings and services—and whether those shifts persist over time.

### Out-of-pocket distribution: predictability gains with uneven ability to benefit

While in a traditional PPO deductible/coinsurance design, out-of-pocket exposure is front-loaded and highly state dependent, dynamic copay plans attempt to replace that volatile function with fixed prices that are knowable in advance and stable across the year for a service/provider. The distribution of benefits is skewed toward members needing “plannable” care; on the other hand, members with complex conditions often face narrower feasible choice sets. Dynamic copays can result in better member experience, only when the description of care can be translated into provider, location, and service specific identifiers.^13^

## Key shortcomings and implementation risks

### Limited relevance for urgent/emergency care and non-shoppable spending

Shopping is not a viable strategy for emergencies, acute exacerbations, or time-sensitive decisions. Even for plannable care, the share of spending that is both shoppable and incurred by members below their MOOP is meaningfully smaller than the commonly cited 40% shoppable estimate.^38^ Members with chronic or complex conditions often face narrower feasible choice sets driven by referral patterns, insurance acceptance, and continuity considerations. Surest has paired its plan with ancillary programs for chronic disease and musculoskeletal care, but those programs operate alongside rather than within the dynamic-copay mechanism, and their effectiveness is a separate empirical question.

### Lack of inherent care coordination

Unlike HMO style gatekeeping or primary care centered models, dynamic copay plans do not, inherently, incentivize having a care coordinator accountable for a patient. Care coordination is widely understood as ensuring that providers and organizations deliver the right care at the right time across settings.^41^ As care coordination is essential to ensure patients receive coherent and continuous care across providers and settings, dynamic copay plans, per se, may fall short because no one takes responsibility for the patient's full treatment plan.

### Data integrity risks: directories and network status remain a difficult problem

The promise of the dynamic copay model depends on being able to correctly identify which provider/location the member will see and whether the provider is truly available in-network. There exists literature on directory discrepancies—Zhu and Barnett in 2025 discuss that CMS report found more than half of clinician listings in Medicare Advantage directories had at least one error, and a 2023 audit of directories from five large national insurers found inconsistencies in 81% of entries.^42^ Empirical work in Affordable Care Act (ACA) marketplace directories also finds that inaccuracies can persist over time, indicating that “bad directory data” is an operational risk.^43^

### Member trust rests on whether the displayed copays are honored at the time of adjudication

A “pay what you see” promise fails the moment displayed copays are not honored at adjudication. Surest's own technical documentation acknowledges that variable copays may return as ranges when the system cannot determine an exact match.^13^ Plans need explicit hold-harmless rules for plan-side mismatches, traceable copay determinations linked to specific contract terms, and rapid-resolution support pathways. Member trust eroded by mismatches is difficult to rebuild and may extend beyond the affected member through word-of-mouth and online channels.

### Service-definition uncertainty

When the correlation of cost-sharing across a cluster of related CPT codes for a given clinician is high, members face limited incidental variance, and the headline copay is informative. When that correlation is low, members face hidden risk that the displayed copay does not capture—for example, when actual coding includes a facility fee or modifier that was not part of the headline service. The empirical correlation is likely to vary by service line and contract and warrants direct measurement rather than assumption.

### Risk selection and employer risk-pool benefits

Consumer-directed plans have historically attracted healthier and more digitally engaged enrollees, raising risk-pool and equity concerns when offered alongside traditional plans.^39,44^ If dynamic copay plans disproportionately attract members who are comfortable shopping through an app, the design may improve member experience for those members while leaving traditional plans with adverse risk pools. Equity analysis must treat digital-access and health-literacy disparities as first-order concerns.

## Discussion

Dynamic copay plans address a real and well-documented friction—members' inability to translate negotiated rates and benefit rules into a comparable, decision-ready price before care. The design's incremental contribution is operational rather than theoretical: finer granularity, earlier salience, and a transparency-data infrastructure that earlier tiered networks lacked. Where the existing tiered-network and reference-pricing literature provide cause for cautious optimism on modest steerage and predictability gains, they also caution against expansive claims about total-cost-of-care effects, durability, or behavioral transformation. The behavioral economics evidence further suggests that even well-designed price displays operate against well-documented limits on consumer shopping, particularly when copay differentials are below approximately $300 or when trusted clinician recommendations point in a different direction.

A layered design—dynamic copays for predictability and intra-tier salience, reference pricing to set a clear maximum employer contribution for shoppable services, and tiered networks to anchor structural value differentiation—plausibly captures more of the available leverage than any single component alone. Such a design must be paired with supply-side incentives that act on the high-cost tail and align provider behavior, since member-facing cost-sharing has limited reach above the OOP maximum and below clinically meaningful copay differentials. The relevant policy question is not whether dynamic copays are superior to tiered networks or VBID but how these tools are most productively combined.

### Implications for plans and third-party administrators

A copay integrity program rests on three commitments. First, data governance prioritizing provider-location-rate linkages with the same rigor as claims-payment accuracy; published copays must be tied to identifiable contract terms and refreshed when contracts change. Second, episode-level logic that aggregates likely ancillary components and surfaces conservative ranges when accuracy cannot be guaranteed. Third, rapid resolution pathways for displayed-vs-paid copay mismatches, with explicit hold-harmless provisions when mismatches are attributable to plan-side data quality. Without these commitments, the design's central promise—that the displayed copay is the paid copay—erodes on first contact with adjudication.

### Implications for employers and plan sponsors

Employers should not evaluate dynamic copay plans on average savings alone. Reporting requirements should include displayed-vs-paid copay agreement, member-experience metrics, and equity disaggregation by digital access and health literacy. Practical guardrails include caps on copay differentials for essential services, explicit hold-harmless provisions for mismatched copays, and contractual commitments to copay refresh frequency. The premium implications of dynamic copay designs depend on whether net-of-administrative-cost claim savings are passed through to plan sponsors; this is a contracting question rather than a benefit-design question, and employers should treat it as such.

### Implications for federal policy and future research

Federal price-transparency rules created the data infrastructure that makes dynamic copays operationally feasible at scale, and they remain a necessary but insufficient condition for member-facing utility.^45^ Independent research is needed in several directions—empirical estimation of the share of spending that is jointly shoppable and below MOOP; selection-corrected analyses of spending and access effects, including methods that account for non-random selection into tiers and into plans; member-experience evaluations of OOP predictability that go beyond average satisfaction; equity analyses by digital access, language, and health literacy; and direct evidence on whether displayed copays are honored at adjudication. In their current form, dynamic copay plans are a meaningful evolution of consumer-directed design rather than its replacement. Their value will depend on whether the underlying operational machinery delivers on the experience they promise, and on whether they are paired with the supply-side, care-coordination, and equity safeguards that the prior two decades of evidence identify as necessary complements.

## Supplementary Material

qxag139_Supplementary_Data
